# Delayed Initiation but Not Gradual Advancement of Enteral Formula Feeding Reduces the Incidence of Necrotizing Enterocolitis (NEC) in Preterm Pigs

**DOI:** 10.1371/journal.pone.0106888

**Published:** 2014-09-19

**Authors:** Nada Ghoneim, Caroline Bauchart-Thevret, Berthe Oosterloo, Barbara Stoll, Madhulika Kulkarni, Miguel Saenz de Pipaon, Irving J. Zamora, Oluyinka O. Olutoye, Brian Berg, Anja Wittke, Douglas G. Burrin

**Affiliations:** 1 Section of Neonatology, Texas Children's Hospital, Division of Pediatric Surgery, Michael E. DeBakey Department of Surgery, Baylor College of Medicine, Houston, Texas, United States of America; 2 United States Department of Agriculture/Agricultural Research Service Children's Nutrition Research Center, Texas Children's Hospital, Division of Pediatric Surgery, Michael E. DeBakey Department of Surgery, Baylor College of Medicine, Houston, Texas, United States of America; 3 Section of Gastroenterology, Hepatology and Nutrition, Department of Pediatrics, Texas Children's Hospital, Division of Pediatric Surgery, Michael E. DeBakey Department of Surgery, Baylor College of Medicine, Houston, Texas, United States of America; 4 Texas Children's Hospital, Division of Pediatric Surgery, Michael E. DeBakey Department of Surgery, Baylor College of Medicine, Houston, Texas, United States of America; 5 Department of Neonatology, La Paz University Hospital, Madrid, Spain; 6 Mead Johnson Pediatric Nutrition Institute, Evansville, Indiana, United States of America; Icahn School of Medicine at Mount Sinai, Argentina

## Abstract

Enteral formula feeding is a risk factor for necrotizing enterocolitis (NEC) in premature infants, yet studies are conflicting regarding the safest timing for introduction and advancement of feeds. Our aim was to test the effects of early vs. late initiation and abrupt vs. gradual advancement of enteral feeding of an intact vs. hydrolyzed protein formula on NEC incidence and severity in preterm pigs. In Experiment 1, preterm pigs received total parenteral nutrition (TPN) at birth with abrupt initiation of enteral formula feeds (50% full intake) on d of life (DOL) 2 (EA) or 5 (LA) while PN continued. Pigs were also fed formula containing either intact or hydrolyzed protein. In Experiment 2, preterm pigs received TPN at birth with enteral, hydrolyzed-protein formula feeds introduced on DOL 2 either abruptly (EA; 50% full feeds) or gradually (EG; 10–50% full feeds over 5 d) while PN continued. NEC incidence and severity were assessed based on macroscopic and histological scoring. In Experiment 1, NEC incidence (41% vs. 70%, *P*<0.05) and severity were reduced in LA vs. EA groups and LA was associated with a higher survival rate, daily weight gain and jejunum villus height. Piglets fed hydrolyzed vs. intact protein formula had lower stomach content weights and similar NEC incidence. In Experiment 2, NEC incidence and severity were not different between pigs the EG vs. EA group. Proinflammatory gene expression (IL-1β, IL-6 and S100A9) in the ileum was lower in both LA and EG vs. EA groups. In conclusion, delayed initiation but not gradual advancement of enteral feeding is protective against NEC in preterm pigs. Feeding hydrolyzed vs. intact protein formula improved gastric transit without affecting the NEC incidence.

## Introduction

Necrotizing enterocolitis (NEC) remains the most common gastrointestinal disease in preterm neonates and has devastating complications. It occurs in 5–10% of very low birth weight (VLBW, <1500 g) infants [1] with a mortality rate of 15–25%, which has not improved appreciably in the past 30 years [2]. The NEC risk has consistently been shown to be inversely related to birth weight and gestational age, most commonly occurring in infants less than 32 weeks or less than 1500 g at birth [3], [4]. While mild cases may lead to delayed feeding and growth with reliance on parenteral nutrition for a defined period, severe cases require surgery with bowel resection and can result in death in 30–40% [2]. Those that survive may end up with short bowel syndrome with lifelong dependence on parenteral nutrition, as well as other complications of surgery such as strictures and infection. These patients are also at risk for adverse neurodevelopmental outcomes [3].

Understanding the pathophysiology of NEC is integral to its prevention. Prematurity and enteral feeding have been shown to be consistent risk factors for NEC [3]. Intestinal immaturity combined with the introduction of enteral feeding and bacterial colonization also contributes to the pathogenesis of the disease [5]. At the molecular level, studies in both humans and animal models have highlighted the important role of the innate immune system in the pathogenesis of NEC. In particular, an exaggerated activation of the toll-like receptor-4 (TLR-4) signaling pathway in the setting of prematurity leads to a heightened inflammatory response with release of interleukins and other inflammatory mediators, leaving the intestinal epithelial barrier susceptible to damage and bacterial translocation [6]–[10]. The addition of enteral feeding in the susceptible host can then lead to sepsis and the development of NEC under certain conditions.

Various feeding strategies for the prevention of NEC have been studied in premature infants. Human milk has consistently been shown to be protective when compared to formula [11]–[13], which is thought to be related to factors in human milk that reduce the inflammatory response and allow for improved gut barrier function [14]. However, clinical studies show conflicting results about the optimal timing of introduction of enteral feeds for NEC prevention. In a review of nine clinical trials involving early trophic feeding (within 96 h of birth) vs. fasting VLBW infants, authors concluded that data were insufficient to inform clinical practice and could not exclude beneficial or harmful effects [15]. Results from another systematic review of seven trials comparing early vs. delayed initiation of progressive enteral feeding suggested that delaying the introduction of progressive enteral feeds beyond four days after birth does not affect the risk of developing NEC in VLBW (including growth-restricted) infants, and may in fact delay establishment of full enteral feeds [16]. While another review from the same group showed that slow advancement of enteral feeds did not reduce the incidence of NEC in VLBW infants [17], another large randomized-controlled trial comparing rapid vs. gradual advancement of feeds was closed early due to development of NEC in a significant number of patients assigned to the rapid advancement group [18]. Ethical considerations, methodological difficulties, genetic heterogeneity, differences in disease and intensive care treatment, and difficulty in obtaining adequate sample sizes limits the study of these different feeding practices in infants and may lead to these discrepancies in results.

The preterm pig offers an effective and relevant animal model for the study of NEC. The neonatal pig has anatomical, physiological, immunological, and metabolic similarities with the human neonate [19], [20]. Previous studies in the preterm pig have shown that formula feeding induces NEC at a higher rate than colostrum [21], and maltodextrin- vs. lactose-based formula increases the risk of NEC [22]. Additionally, previous preterm pig studies showed that delaying enteral formula feeds while providing total parenteral nutrition (TPN) increased the incidence of NEC once feeds were introduced [23]. In most of these previous studies, preterm pigs were transitioned abruptly to enteral feeding and withdrawal of TPN support. This does not reflect the common clinical practice in preterm infants, which involves continuing parenteral nutrition (PN) while enteral feeds are gradual advanced over a period of days. Additionally, while it has been shown in clinical studies that hydrolyzed vs. intact protein formula improves digestion and intestinal transit, the effect on the incidence of NEC has not been studied.

The aim of this study was to measure the incidence and severity of NEC in preterm pigs via the following feeding regimens while providing a clinically-relevant scenario with continuation of parenteral nutrition: (1) *Experiment 1*: early vs. delayed initiation of enteral formula feeds composed of hydrolyzed vs. intact protein, and (2) *Experiment 2*: gradual versus abrupt advancement of enteral hydrolyzed protein formula feeding. We hypothesized that, based on the results of our previous work in the preterm pig, a prolonged period of PN prior to initiation of feeds would increase the incidence and severity of NEC. We also hypothesized that gradual advancement of feeding would reduce the incidence and severity of NEC.

## Materials and Methods

The protocol was approved by the Animal Protocol Review Committee of Baylor College of Medicine and conducted in accordance with the Guide for the Care and Use of Laboratory Animals [Department of Health and Human Services publication no. 85–23, revised 1985, Office of Science and Health Reports, NIH, Bethesda, MD]. All surgeries were performed under general anesthesia, and all efforts were made to minimize suffering.

### Animals and surgery

Pregnant crossbred sows were obtained from the Texas Department of Criminal Justice (Huntsville, TX). Sows were housed in the Children's Nutrition Research Center and were given food and water ad libitum. Preterm pigs were delivered by cesarean section at 103 days of gestation (115 days term). Before cesarean section, sows were first injected intramuscularly with glycopyrrolate (0.01 mg/kg; Baxter Healthcare Corp., Deerfield, IL) followed by a mixture of ketamine (20 mg/kg) + xylazine (2 mg/kg; Butler Schein, Dublin, OH). Then, 40–50 mL of Lidocaine (2%; Sparhawk Laboratories, Inc., Lenexa, KS) was injected along the lumbar vertebrae to induce paravertebral blockade. After endotracheal intubation, anesthesia was maintained with isoflurane inhalation (1–3% in oxygen). Under sterile conditions, a midline laparotomy incision was made and the uterus exposed. Following a hysterotomy incision on the anti-mesenteric uterine surface, preterm pigs were sequentially removed after ligating and transecting the umbilical cord and immediately placed in cages housed at 31–32°C. After delivery, pigs underwent surgery for placement of jugular catheters for TPN administration and orogastric (OG) feeding tubes. Following surgery on day 0, pigs were started on TPN at 5 mL/kg*h (120 mL/kg*d or 50% of total daily requirement). TPN at this rate provided (per kg/d): 410 kJ energy, 12.5 g dextrose, 6.5 g amino acids, 2.5 g fat (Intralipid 20%; Fresenius Kabi, Bad Homburg, Germany). During the first 24 h, maternal plasma (16 mL/kg) was administered for passive immunological protection. On day 1, TPN was increased to 6 mL/kg*h.

### Study design: Experiment 1 (Early vs. Late Feeding)

**Figure 1:**
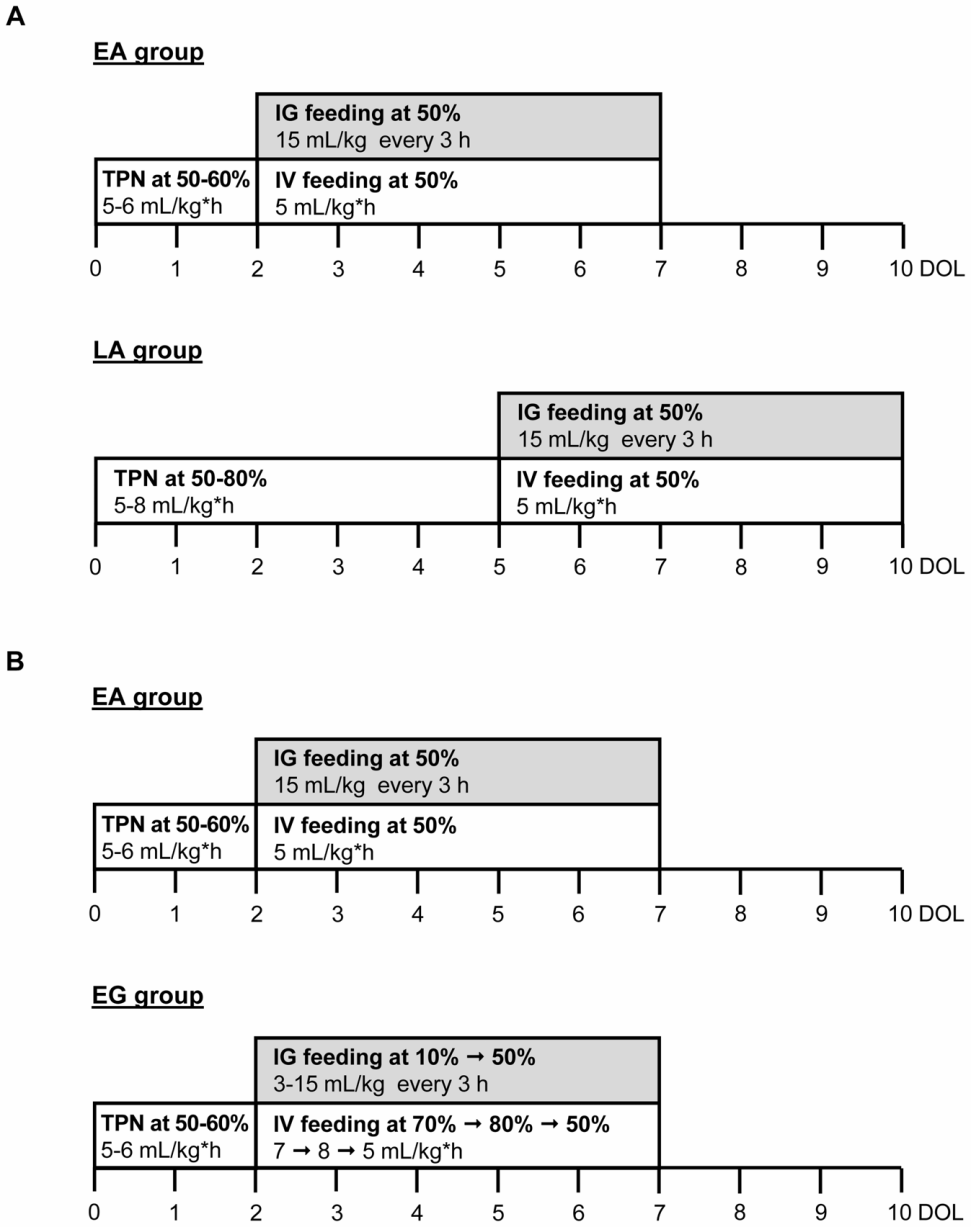
Study design of Experiment 1, EA vs. LA, and Experiment 2, EA vs. EG. At DOL 0, pigs were delivered 12-d preterm via C-section and were surgically implanted with orogastric and jugular catheters. (A) In Experiment 1, enteral formula (IG feeding with intact or hydrolyzed protein) was introduced abruptly at 50% of their total feeding intake either on DOL 3 after 2 days of TPN (EA group), or on DOL 6 after 5 days of TPN (LA group). (B) In Experiment 2, enteral formula (IG feeding with hydrolyzed casein) was introduced either abruptly (EA group) at 50% of their total feeding intake on DOL 3 after 2 days of TPN, or advanced gradually (EG group) starting at 10% of their total feeding intake on DOL 3 up to 50% on DOL 7 with a 10%-daily increase. Pigs were weighed every other day and their intake adjusted accordingly. Signs of NEC were monitored at each feed. Pigs were euthanized at signs of NEC or 5 d after start of IG feeding and tissues were collected. EA, early abrupt; LA, late abrupt. EG, early gradual.

A total of 62 pigs from 9 litters were included in this experiment. Based on body weight, pigs were randomly assigned to Early Abrupt (EA) or Late Abrupt (LA) introduction of enteral feeds. Pigs in the EA group (n = 40) and pigs in the LA group (n = 22) began enteral feeds (IG feeding) on day of life (DOL) 2 and DOL 5, respectively, at 50% total daily requirement, and PN (IV feeding) was continued at 50% to provide a total of 100% daily nutritional needs (**Figure 1A**). Bolus feeds were given every 3 h via the OG tube 8 times per day. The formula was designed to contain the same ingredient profile as that of preterm infant formula, but with increased protein and fat to meet the nutrient requirements of piglets. The formula carbohydrate component was corn-syrup solids based on our previous findings that this induces NEC in preterm pigs [22] (**Table S1**). Pigs in each treatment group were given either hydrolyzed (EA, n = 27; LA, n = 11) or intact protein formulas (EA, n = 13; LA, n = 11). The main difference between the hydrolyzed and intact protein formula was the use of hydrolyzed casein rather than intact casein. It is important to note that the whey protein fractions were not hydrolyzed.

Pigs were weighed every other day and their intake was adjusted accordingly. At the time of each feeding, pigs were checked for clinical symptoms of NEC including presence of gastric residuals, vomiting, diarrhea, bloody stools, abdominal distension, and respiratory distress. Pigs were euthanized when they developed signs of NEC or after 5 days of enteral feeds (DOL 7 for EA group and DOL 10 for LA group).

### Study design: Experiment 2 (Abrupt vs. Gradual Feeding)

A total of 43 pigs from 5 litters were included in Experiment 2. Based on body weight, pigs were randomly assigned to EA (Early Abrupt) or EG (Early Gradual) introduction of enteral feeds. Pigs in the EA group (n = 27) received the same feeding and TPN protocol as in Experiment 1; however, only hydrolyzed protein formula was used. Pigs in the EG group (n = 16) also began enteral feeds (IG feeding) of hydrolyzed protein formula on DOL 2 but were started at 10% total daily requirement (3 mL/kg every 3 h), and PN (IV feeding) at 70% (7 mL/kg*h). Each subsequent day, the IG feeding rate was increased by 10% to a maximum of 50% total daily requirement by DOL 6, and PN was adjusted accordingly to maintain 100% total daily nutritional needs (**Figure 1B**). As in Experiment 1, pigs were weighed every other day and intakes were adjusted accordingly, and pigs were monitored for signs of NEC every 3 h at the time of each feed. Pigs were euthanized when they developed signs of NEC or after 5 days of enteral feeds (DOL 7 for both groups).

### Tissue analysis and NEC evaluation

At the time of death, each pig was weighed and necropsy was performed. Stomach contents were weighed, and stomach, jejunum, ileum, and colon were inspected for signs of NEC. Each of these areas was assigned a clinical NEC severity score (1–6) based on established guidelines from previous studies [24]. Scores of 5 or 6 were given if gross necrosis and/or pneumatosis were present. Pigs with a clinical NEC severity score of 3 or greater in any of the 4 tissues were given a preliminary diagnosis of NEC.

Samples of intestinal tissue (jejunum, ileum, and colon) were collected and frozen at −80°C for gene expression analysis and fixed in 10% formalin for histological analyses. Formalin-fixed sections were embedded in paraffin, cut into 4 µm-thick sections and stained with H&E. These sections were used for morphometric measurements (villus height and crypt depth) using Scion Image Software (Scion Corporation 2000–2001, version Alpha 4.0.3.2, Maryland, USA) and histological NEC scoring. Histological NEC scoring was based on a previously established scoring system for NEC developed in the rodent model [25]. Intestinal tissues were inspected microscopically and assigned a histological NEC severity score (0–4) based on the degree of epithelial and/or mucosal damage. Tissues with greater damage received scores of 3–4 indicating the presence of necrosis and/or pneumatosis. The observer was blinded to the preliminary NEC diagnosis of each pig that had been assigned by clinical scoring. Pigs with a histological NEC severity score of 2 or greater were given a definitive diagnosis of NEC. Bright field images of histological cross sections of the jejunum, ileum, and colon stained with H&E were taken using Picture Frame Application 2.3 software.

### Real-time quantitative PCR of ileal tissue

Real-time quantitative PCR (RT-qPCR) analysis was performed on ileal samples of all pigs to evaluate for expression of the following genes involved in innate immunity and implicated in NEC development: IL1-β, IL-6, S100A9, TLR-4, TNFα. Total RNA was isolated using the RNeasy Midi kit (Qiagen) and was DNAse-treated using the DNase-free kit from Ambion (#AM1906). The DNAse-treated RNA was used for the reverse transcription reactions using the High Capacity cDNA Reverse Transcription kit (Applied Biosystem), and cDNA products were stored at 4°C until RT-qPCR was performed using Superscript III Platinum Two-Step qPCR kit with SYBR Green (Invitrogen) on Bio Rad CFX96 real-time PCR machines. Oligonucleotide primer sequences were designed using NCBI Primer Blast (**Table S2**). Relative quantification of target mRNA expression was calculated and normalized to β-actin expression. All reactions were performed under the following thermal cycling conditions: 10 minutes at 95°C, followed by 40 cycles of 95°C for 15 seconds and 60°C for 60 seconds. The 2^−ΔΔCT^ method was used to compare gene expression levels between samples, which were analyzed to determine the fold induction of mRNA expression.

### Statistical analysis

Data were analyzed using Stata Version 12 software (College Station, Texas), Graph-Pad Prism version 5.02 software (GraphPad Software, Inc.), and Minitab Release 14.20 software (Minitab Inc.). NEC incidence based on diagnosis made by clinical or histological scoring was compared among pigs in the EA and LA groups, EA and EG groups, as well as in pigs receiving hydrolyzed vs. intact protein formula using the Fisher's Exact test. In Experiment 1, a binary logistic regression was performed on overall NEC incidence data with Dietary treatment (EA vs. LA) and Nutrition (intact vs. hydrolyzed protein) as main effects. Clinical and histological NEC severity scores were compared using the Wilcoxon Rank Sum test for ordinal data. Clinical symptom occurrence for NEC was calculated for each day and analyzed by ANOVA for repeated measures using a general linear model with Dietary treatment and Day as main effects. Kaplan-Meier survival analysis and the log-rank test were used to evaluate the overall survival after the initiation of the first feeds. A linear regression analysis was performed to examine the effect of clinical and histological NEC severity scores on survival. RT-qPCR data from Experiment 1 were analyzed using two-way ANOVA for multiple comparisons with Dietary treatment (EA vs. LA) and Nutrition (intact vs. hydrolyzed protein) as main effects. All other results were analyzed using the Student's T-test. All data are expressed as means ± SEM. Differences were considered to be statistically significant at *P*<0.05.

## Results

### Experiment 1 (Early vs. Late Feeding)

#### Birth weight and body weight gain

Pigs in the EA and LA groups with intact and hydrolyzed protein formula combined had similar birth weights (**Table 1**). However, pigs fed with hydrolyzed protein formula had a smaller birth weight than pigs fed the intact protein formula in the LA group (**Table 1**). Daily weight gain over the course of the study was greater (+28%, *P*<0.05) in the LA group than in the EA group. However, this increase in body weight gain was only significant in pigs fed the intact protein formula (+42% LA vs. EA, *P*<0.05) (**Table 1**).

**Table 1:** Birth weight and body weight gain in pigs in Experiments 1 and 2.

	Birth weight	Birth weight range	Body weight gain
	*g*	*g*	g/kg*d
**Experiment 1**			
EA (n = 40)	1098±40	500–1621	43±4
Intact protein (n = 13)	1224±64	790–1621	38±6
Hydrolyzed protein (n = 27)	1036±47	500–1526	46±5
LA (n = 22)	1107±49	709–1452	55±2*
Intact protein (n = 11)	1234±59	817–1452	54±3*
Hydrolyzed protein (n = 11)	979±59^††^	709–1238	56±3
**Experiment 2**			
EA (n = 27)	1036±47	500–1526	46±5
EG (n = 16)	1099±60	607–1515	44±8

Values are means ± SEM. *P<0.05 EA vs. LA; ^††^ *P*<0.01 Intact protein vs. Hydrolyzed protein. EA, early abrupt; LA, late abrupt; EG, early gradual.

#### NEC incidence and severity scores and NEC clinical symptoms

**Figure 2:**
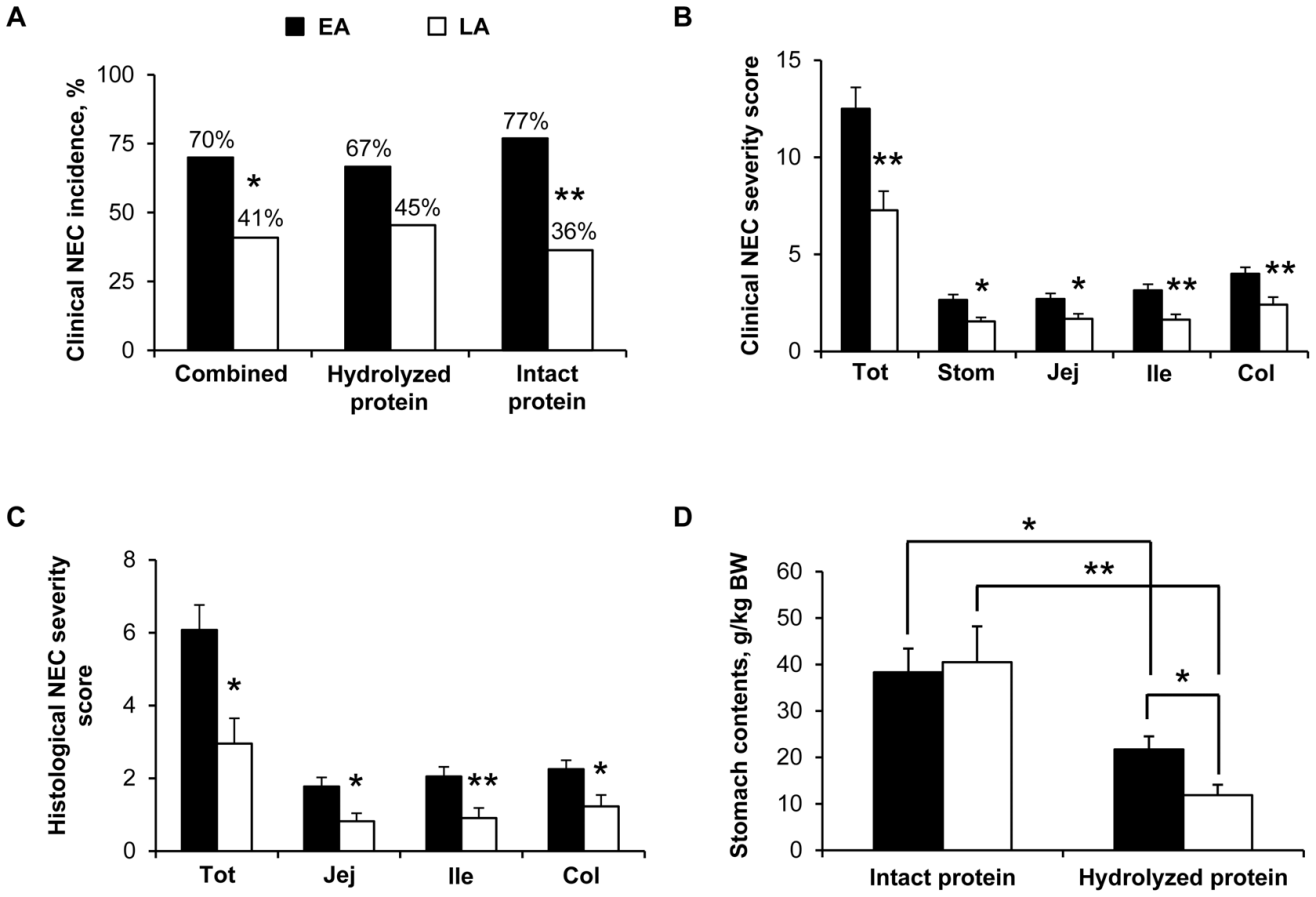
Clinical NEC incidence, NEC severity scores, and stomach content weights in Experiment 1. (A) Clinical NEC incidence values are the sum of all clinical NEC cases and expressed as percentage. Clinical NEC incidence was the same as histological NEC incidence. Combined, n = 22 (LA)-40 (EA); Hydrolyzed protein, n = 11 (LA)-27 (EA); Intact protein, n = 11 (LA)-13 (EA); *P<0.05, **P<0.01 EA vs. LA. (B) Clinical NEC severity scores determined in the stomach (Stom), jejunum (Jej), ileum (Ile), colon (Col) and all those 4 tissues combined (Tot). For each tissue, grades 1–2 represent “No NEC” and grades 3–6 represent “NEC”. For combined tissues, the maximum clinical NEC severity score is 24. EA, n = 40; LA, n = 22; *P<0.05, **P<0.01 EA vs. LA. (C) Histological NEC severity scores determined in the jejunum (Jej), ileum (Ile), colon (Col) and all those 3 tissues combined (Tot). For each tissue, grades 0–1 represent” No NEC” and grades 2–4 represent “NEC”. For combined tissues, the maximum histological NEC severity score is 12. EA, n = 40; LA, n = 22; *P<0.05, **P<0.01 EA vs. LA. (D) Stomach content weight values are expressed as g/kg of body weight (BW). Intact protein, n = 8 (EA)-10 (LA); Hydrolyzed protein, n = 10 (LA)-22 (EA); *P<0.05, **P<0.01 EA vs. LA. EA, early abrupt; LA, late abrupt.

The incidence of NEC determined by clinical NEC severity scores was the same as that determined by histological NEC severity scores in both groups (i.e. 70% in EA and 41% in LA). The incidence of NEC was lower (−41%, *P*<0.05) in the LA group than in the EA group; in pigs fed the intact protein formula (−53% LA vs. EA, *P*<0.01) (**Figure 2A**). There was no significant difference in the incidence of NEC in pigs that received hydrolyzed protein formula (67% in EA, 45% in LA) compared to those that received intact protein formula (77% in EA, 36% in LA). The NEC severity was assessed in each group using both clinical and histological scoring systems. Clinical and histological NEC severity scores were lower (−38% to −56%, *P*<0.05) in the LA group than in the EA group, both overall and in the individual tissues (**Figure 2B–C**). There was no difference between hydrolyzed vs. intact protein formula on clinical (EA, 12.59±1.43 vs. 12.31±1.71; LA, 6.73±1.32 vs. 7.82±1.51) and histological (EA, 5.89±0.87 vs. 6.46±1.14; LA, 2.73±0.97 vs. 3.18±1.03) NEC severity scores, respectively. Over the 5-d enteral feeding period, gastric residuals were present in about 40% to 70% of the pigs without effect of Dietary treatment and Day (data not shown). Vomiting rate was less than 10% of the pigs (data not shown). Diarrhea was evident in about 60% of the pigs after the second day of enteral feeding and stayed at about the same rate until the end of the enteral feeding without any difference between the treatment groups (**Figure S1A**). Bloody stools occurred in about 10% of the pigs without Dietary treatment effect (data not shown). Abdominal distension was evident in up to 30–45% of the pigs after 2–3 days of enteral feeding without any difference between the 2 treatment groups (**Figure S1B**) but with a high occurrence (up to 90% in the EA group, **Figure S1C**; up to 55% in the LA group, data not shown) with the intact protein diet compared to the hydrolyzed protein diet (occurrence up to 20% in both EA and LA groups; **Figure S1C** for the EA group, data not shown for the LA group) (*P*<0.001, intact vs. hydrolyzed protein). Respiratory distress occurred in about 20% to 30% of the pigs driven by the Intact protein diet (*P*<0.001, intact vs. hydrolyzed protein) (**Figures S1D–E**). Among the different NEC clinical symptoms checked, bloody stool was the most specific sign for NEC incidence (specificity of 93% with a positive predictive value of 92%, *P*<0.01) and the presence of gastric residuals and abdominal distention were the most sensitive signs for NEC incidence (90% sensitivity for both).

#### Intestinal morphometry and stomach content weight

**Figure 3:**
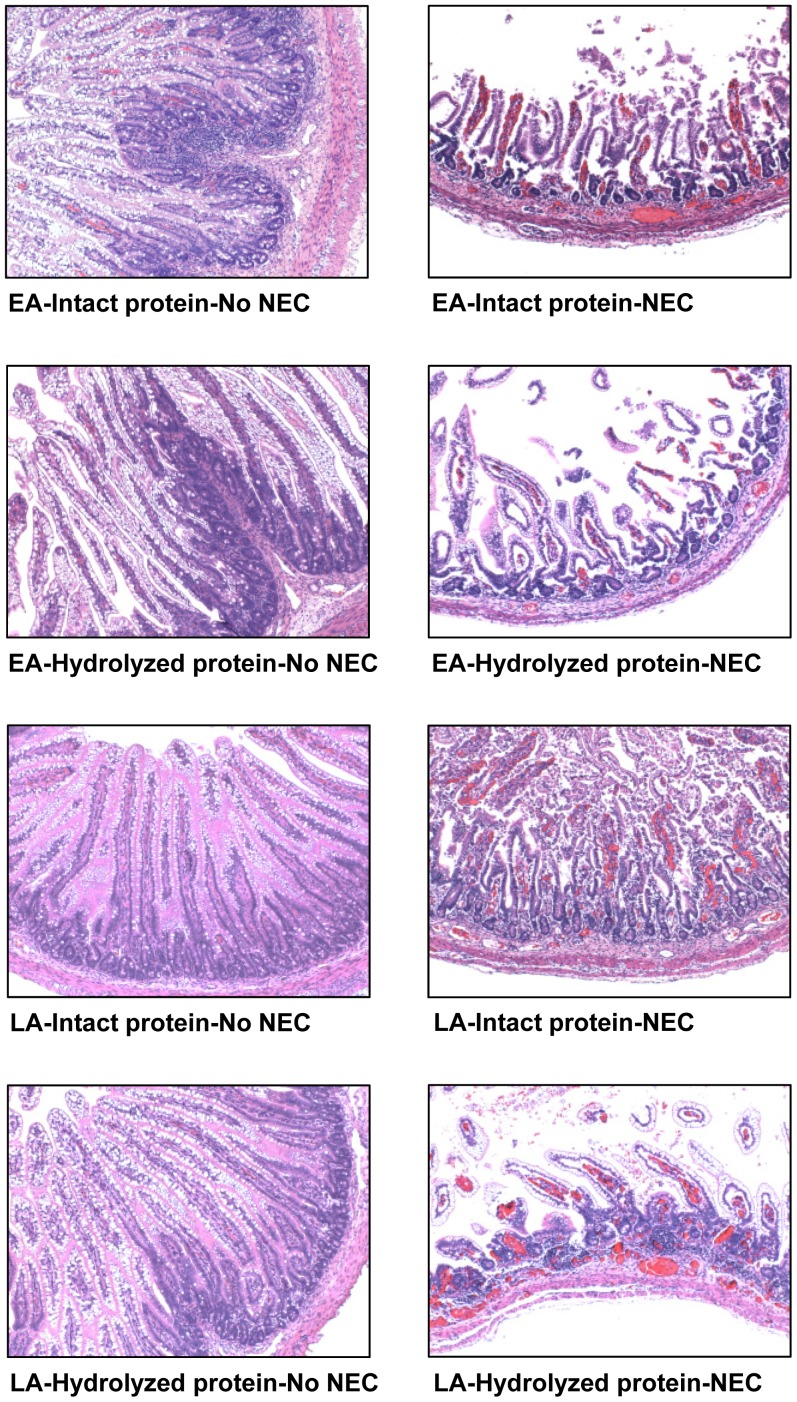
Intestinal cross sections from Experiment 1. Histological cross sections stained with H&E of the ileum from pigs fed either an intact or hydrolyzed protein formula without NEC (No NEC) or that had developed NEC (NEC). All images are presented at 10X magnification. EA, early abrupt; LA, late abrupt.

Morphometric analyses of intestinal samples from jejunum, ileum, and colon were performed. Villus height was higher (+59%, *P*<0.05) in the LA vs. EA group in the jejunum of pigs fed intact protein formula that got NEC (NEC) (**Table 2** **and Figure S2A**). Moreover, in the EA group, jejunal and ileal villus height was lower (−38% to −56%, *P*<0.05) in pigs that got NEC (NEC) compared to pigs that did not get NEC (No NEC) regardless of the type of formula given (**Table 2** **, Figures S2A and 3**). In the LA group, ileal villus height was lower (−54%, *P*<0.01) in NEC vs. No NEC pigs that had been fed intact protein formula (**Table 2** **and** **Figure 3**). Crypt depth was higher (+12%, *P*<0.01) in the LA group compared to the EA group in the jejunum of pigs fed the intact protein formula that did not get NEC (No NEC) (**Table 2** **and Figure S2A**). In addition, crypt depth was also higher (+56%, *P*<0.01) in the LA vs. EA group in the colon of pigs fed the hydrolyzed protein formula that got NEC (NEC) (**Table 2** **and Figure S2B**). In the EA group, jejunal, ileal, and colonic crypt depth was lower (−29% to −41%, *P*<0.01) in NEC vs. No NEC pigs that had been fed hydrolyzed protein formula (**Table 2** **, Figures S2 and 3**).

**Table 2:** Intestinal morphometry in Experiment 1.

	EA		LA	
	Intact	Hydrolyzed	Intact	Hydrolyzed
	(n = 13)	(n = 27)	(n = 11)	(n = 11)
	*µm*			
**Villus height**				
Jejunum				
No NEC	598±60^†^	583±48^††^	626±37	583±67
NEC	350±54	359±46	558±41*	477±133
Ileum				
No NEC	718±54^††^	845±45^†††^	759±68^††^	633±112
NEC	356±65	368±78	349±59	625±161
**Crypt depth**				
Jejunum				
No NEC	85±3	114±7^††^	95±3*	93±4*
NEC	82±3	81±3	92±4	76±19
Ileum				
No NEC	90±3	121±7^†††^	96±2	98±6*
NEC	82±3	77±6	95±5	90±6
Colon				
No NEC	245±13	253±7^†††^	261±5	247±25
NEC	214±16	150±20	147±58	234±22*

Values are means ± SEM. “No NEC” or “NEC” was based on the histological NEC incidence. *P<0.05 EA vs. LA; ^†^ *P*<0.05, ^††^ *P*<0.01, ^†††^ *P*<0.001 NEC vs. No NEC. EA, early abrupt; LA, late abrupt.

Stomach content weights were compared in pigs given hydrolyzed vs. intact protein formula. Stomach contents were greater (+76% in EA, *P*<0.05; +240% in LA, *P*<0.01) in pigs fed intact protein formula than those fed hydrolyzed protein formula (**Figure 2D**). Among pigs fed hydrolyzed protein formula, stomach content weights were lower (−45%, *P*<0.05) in pigs in the LA group than those in the EA group (**Figure 2D**). The slower gastric emptying rate in the pigs fed intact protein formula was also evident in increased abdominal distension and respiratory distress without any evidence of NEC; this adverse effect of intact protein on stomach clotting and respiratory distress is why we used only hydrolyzed protein in Experiment 2.

#### Survival analysis

**Figure 4:**
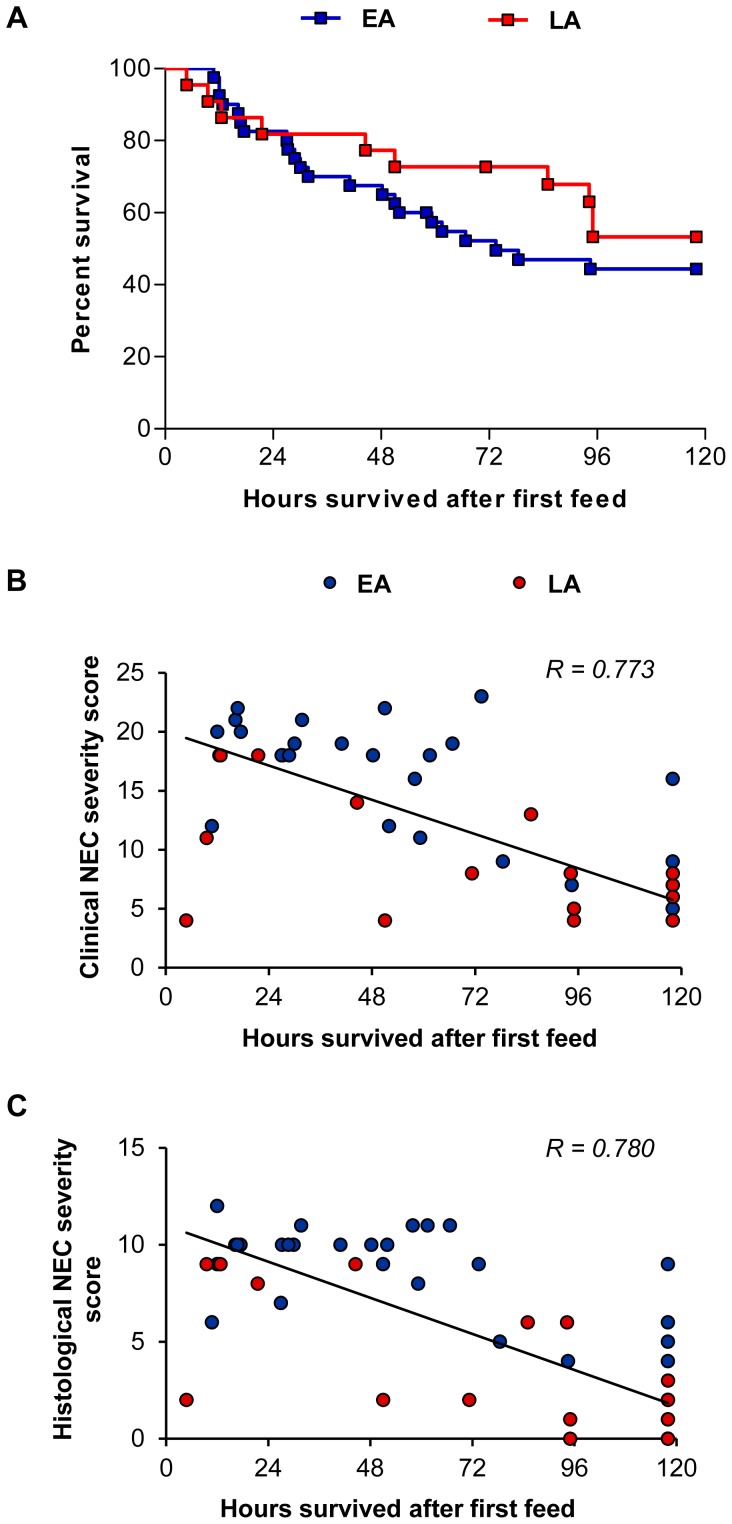
Survival analysis and linear regression analysis of NEC severity scores vs. survival in Experiment 1. (A) Kaplan-Meier survival estimates determined after the first IG feeding and expressed as percentage. Results were not significant. EA, n = 40; LA, n = 22. (B) Linear regression analysis of clinical NEC severity scores vs. survival after the first IG feeding performed on combined EA and LA groups. For each pig, the clinical NEC severity score represents the sum of clinical NEC severity scores (total score) in stomach, jejunum, ileum and colon. Clinical NEC severity scores were negatively correlated with survival (n = 62; *P*<0.001, R = 0.773). (C) Linear regression analysis of histological NEC severity scores vs. survival after the first IG feeding performed on combined EA and LA groups. For each pig, the histological NEC severity score represents the sum of clinical NEC severity scores (total score) in jejunum, ileum and colon. Histological NEC severity scores were negatively correlated with survival (n = 62; *P*<0.001, R = 0.780). EA, early abrupt; LA, late abrupt.

Hours from time of initial feed until death were compared between pigs in the LA vs. EA groups. Pigs in the LA and EA groups had similar survival time (**Figure 4A**), however, pigs that had lower clinical and histological NEC severity scores had improved survival (**Figure 4B–C**).

#### Proinflammatory gene expression

**Figure 5:**
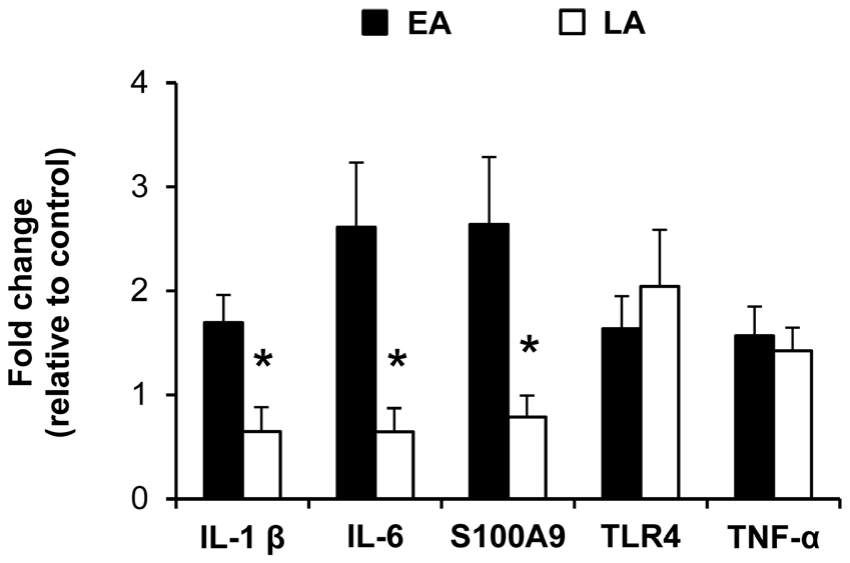
Proinflammatory gene expression in the ileum of pigs in Experiment 1. Gene expression values are expressed as fold change vs. the EA–Intact protein group (No NEC + NEC). β-actin was used as internal control. n = 19–39; *P<0.05 EA vs. LA. EA, early abrupt; LA, late abrupt.

Proinflammatory gene expression in the ileum was compared between all pigs (NEC and NO NEC pigs combined) using a two-way ANOVA with Dietary treatment (EA vs. LA) and Nutrition (intact vs. hydrolyzed protein) as main effects. Since no significant effect of Nutrition was found, gene expression of IL1-β, IL-6, S100A9, TLR-4 and TNF-α in the LA vs. EA groups is shown in **Figure 5** with the intact and hydrolyzed protein groups combined. Pigs in the LA group had lower (−62% to −75%, *P*<0.05) ileal gene expression of IL1-β, IL-6 and S100A9 compared to the EA group (**Figure 5**).

### Experiment 2 (Abrupt vs. Gradual Feeding)

#### Birth weight and body weight gain

Birth weight and body weight gain were similar in the EA and EG groups (**Table 1**).

#### NEC incidence and severity scores and NEC clinical symptoms

**Figure 6:**
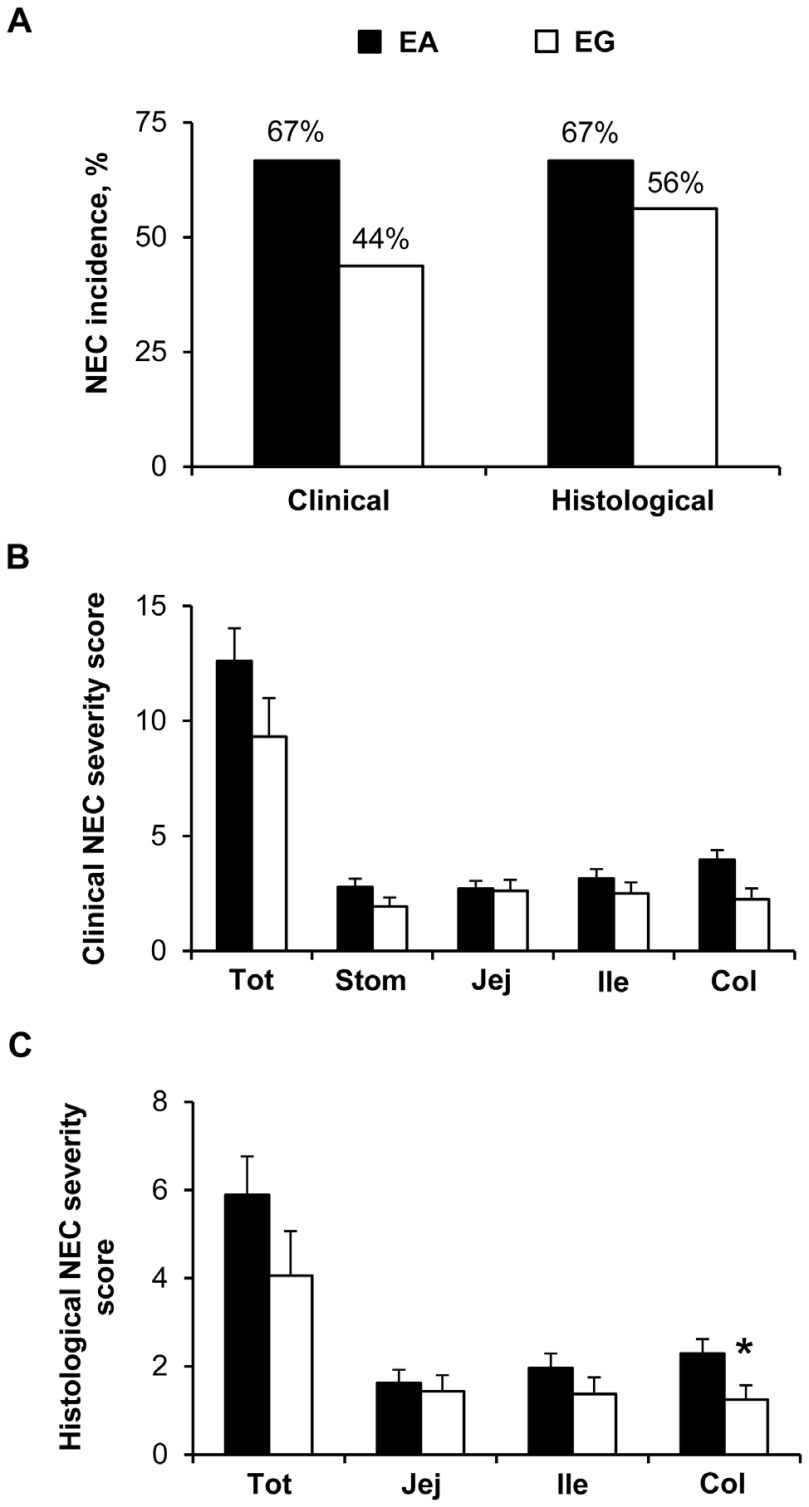
Clinical and histological NEC incidence and NEC severity scores in Experiment 2. (A) NEC incidence values are the sum of all clinical or histological NEC cases and expressed as percentage. Results were not significant. EA, n = 27; EG, n = 16. (B) Clinical NEC severity scores determined in the stomach (Stom), jejunum (Jej), ileum (Ile), colon (Col) and all those 4 tissues combined (Tot). For each tissue, grades 1–2 represent “No NEC” and grades 3–6 represent “NEC”. For combined tissues, the maximum clinical NEC severity score is 24. EA, n = 27; EG, n = 16. (C) Histological NEC severity scores determined in the jejunum (Jej), ileum (Ile), colon (Col) and all those 3 tissues combined (Tot). For each tissue, grades 0–1 represent “No NEC” and grades 2–4 represent “NEC”. For combined tissues, the maximum histological NEC severity score is 12. EA, n = 27; EG, n = 16; *P<0.05 EA vs. EG. EA, early abrupt; EG, early gradual.

NEC incidence by clinical and histological scoring was the same for the EA group (67%) (**Figure 6A**). However, NEC incidence by histological scoring was greater (56% vs. 44%) than by clinical scoring for the EG group (**Figure 6A**). The incidences of both clinical and histological NEC were not different among the EG and EA groups (**Figure 6A**). NEC severity was also lower in the EG group overall and in the individual tissues but only statistically significant in the colon (−31%, *P*<0.05) by histological score (**Figure 6B–C**). At the beginning of the enteral feeding, gastric residuals were present in about 40% to 60% of the pigs and increased up to about 70% at d 4 (Day effect, *P*<0.05) in both groups without Dietary treatment effect (**Figure S3A**). As observed in Experiment 1, vomiting rate was less than 10% of the pigs over the 5-d enteral feeding period (data not shown). Diarrhea incidence was delayed in the EG group compared to the EA group and peaked (55–60% of the pigs) at d 4 in the EG vs. d 2 in the EA group (Dietary treatment effect, *P*<0.001; Day effect, *P*<0.001) (**Figure S3B**). Bloody stool was evident in about 10% or less of the pigs in the EA group, but almost absent in the EG group (Dietary treatment effect, *P*<0.05) (data not shown). Abdominal distension occurred in about 20–30% or less of the pigs without any difference between the two treatment groups (**Figure S3C**). Respiratory distress was evidenced in about 10% or less of the pigs without Dietary treatment effect (data not shown).

#### Intestinal morphometry

Morphometric analysis showed no significant differences in villus height and crypt depth in both the jejunum and the ileum in the EA group vs. EG group (**Table 3**, **Figures S4A** **and** **7**). However, crypt depth in the colon was higher (+61%, *P*<0.01) in the EG group compared to the EA group that got NEC (NEC) (**Table 3** **and** **Figure S4B**). In addition, villus height and crypt depth were shorter (−29% to −68%, *P*<0.01) in pigs that got NEC (NEC) compared to pigs that did not get NEC (No NEC) in both the jejunum and ileum in both the EA and EG groups and in the colon only in the EA group (**Table 3**, **Figures S4** **and** **7**).

**Table 3:** Intestinal morphometry in Experiment 2.

	EA	EG
	(n = 27)	(n = 16)
	*µm*	
**Villus height**		
Jejunum		
No NEC	583±48^††^	682±84^††^
NEC	359±46	303±50
Ileum		
No NEC	845±45^†††^	865±56^†††^
NEC	368±78	280±62
**Crypt depth**		
Jejunum		
No NEC	114±7^††^	124±8^††^
NEC	81±3	87±4
Ileum		
No NEC	121±7^†††^	133±6^††^
NEC	77±6	85±13
Colon		
No NEC	253±7^†††^	258±5
NEC	150±20	241±19**

Values are means ± SEM. “No NEC” or “NEC” was based on the histological NEC incidence. **P<0.01 EA vs. EG; ^††^ *P*<0.01, ^†††^ *P*<0.001 NEC vs. No NEC. EA, early abrupt; EG, early gradual.

**Figure 7:**
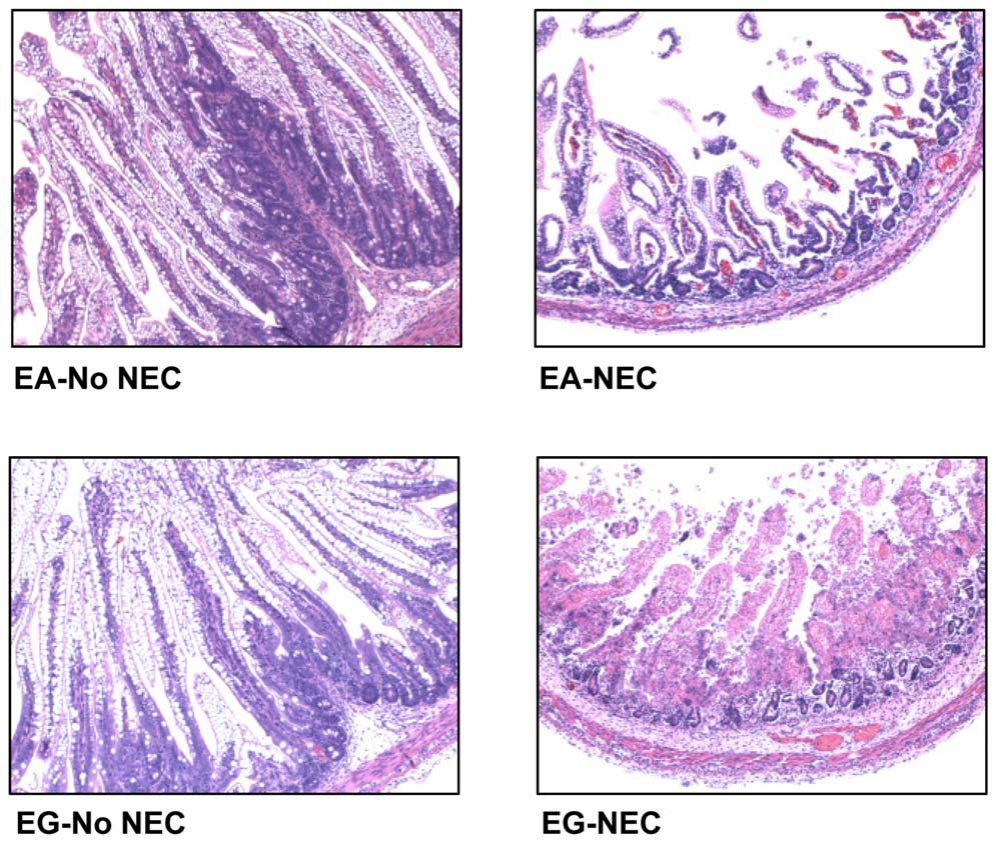
Intestinal cross sections from Experiment 2. Histological cross sections stained with H&E of the ileum in pigs without NEC (No NEC) or that had developed NEC (NEC). All Images are presented at 10X magnification. EA, early abrupt; EG, early gradual.

#### Survival analysis

**Figure 8:**
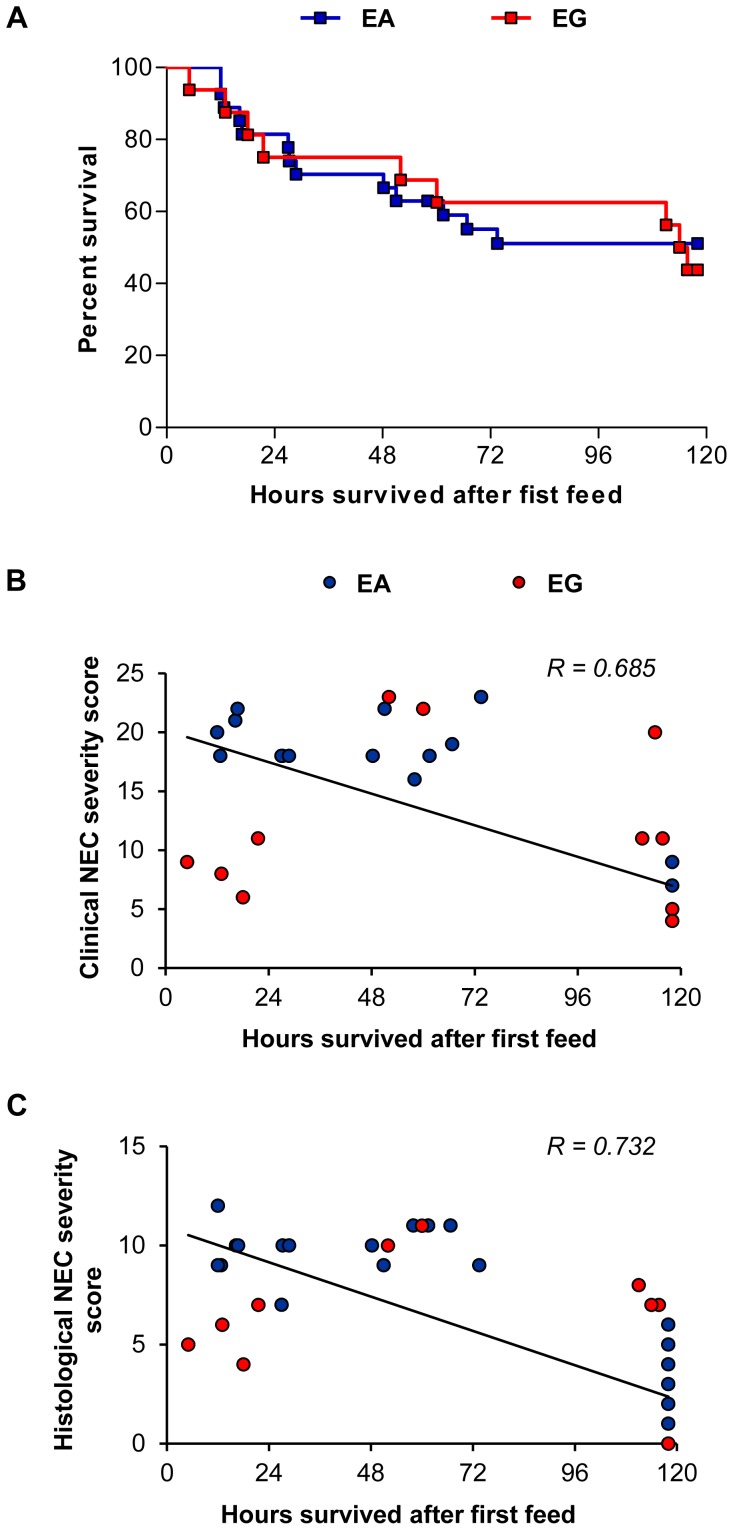
Survival analysis and linear regression analysis of NEC severity scores vs. survival in Experiment 2. (A) Kaplan-Meier survival estimates determined after the first IG feeding and expressed as percentage. Results were not significant. EA, n = 27; EG, n = 16. (B) Linear regression analysis of clinical NEC severity scores vs. survival after the first IG feeding performed on combined EA and EG groups. For each pig, the clinical NEC severity score represents the sum of clinical NEC severity scores (total score) in stomach, jejunum, ileum and colon. Clinical NEC severity scores were negatively correlated with survival (n = 43; *P*<0.001, R = 0.685). (C) Linear regression analysis of histological NEC severity scores vs. survival after the first IG feeding performed on combined EA and EG groups. For each pig, the histological NEC severity score represents the sum of clinical NEC severity scores (total score) in jejunum, ileum and colon. Histological NEC severity scores were negatively correlated with survival (n = 43; *P*<0.001, R = 0.732). EA, early abrupt; EG, early gradual.

Kaplan-Meier survival analysis showed that survival was not different between the pigs in the EG vs. EA group (**Figure 8A**). However, as was observed in Experiment 1, the pigs in Experiment 2 with a high clinical and histological NEC severity score had lower survival (**Figure 8B–C**).

#### Proinflammatory gene expression

**Figure 9:**
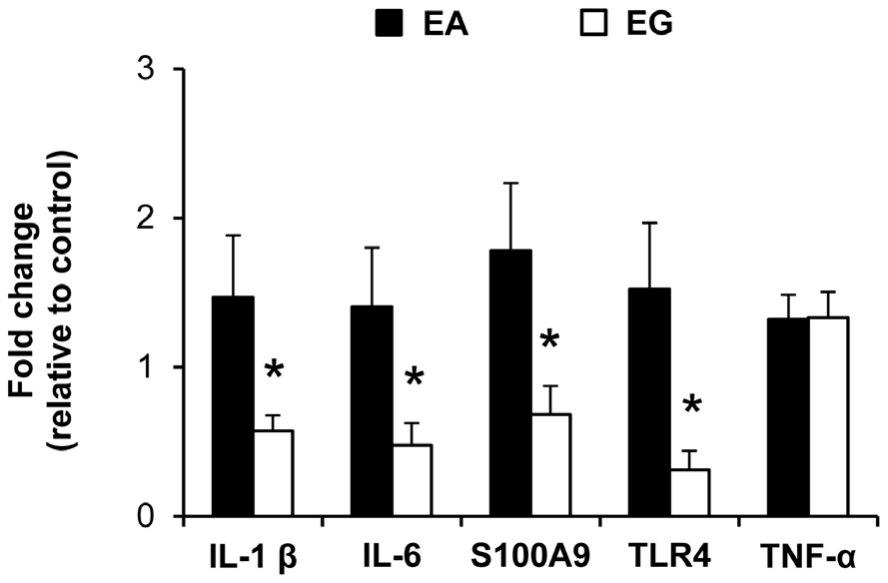
Proinflammatory gene expression in the ileum of pigs in Experiment 2. Gene expression values are expressed as fold change vs. the EA–Hydrolyzed protein group (No NEC + NEC). β-actin was used as internal control. n = 12–27; *P<0.05 EA vs. EG. EA, early abrupt; EG, early gradual.

Proinflammatory gene expression in the ileum of IL1-β, IL-6, S100A9, TLR-4 and TNF-α was compared between all pigs (NEC and NO NEC pigs combined) in the EA vs. EG groups (**Figure 9**). Pigs in the EG group had lower (−61% to −80%, *P*<0.05) ileal gene expression of IL1-β, IL-6, S100A9 and TLR-4 compared to the EA group (**Figure 9**).

## Discussion

The aim of this study was to determine the impact of timing of introduction and rate of advancement of enteral feeding on the incidence and severity of NEC in preterm piglets. The first major finding in this study is that delayed initiation of enteral feeding for 3 days (from DOL 2 to DOL 5) was protective against NEC in the preterm pig. Our results showed that piglets in the LA vs. EA group had lower NEC incidence and lower NEC severity scores throughout the digestive tract which was associated with a higher survival rate, daily weight gain and villus height in the jejunum. This finding was contrary to our hypothesis that prolonged TPN would lead to deterioration of epithelial integrity and thus increase NEC risk [23]. This result may be explained by the fact that, in both EA and LA groups, PN was continued when enteral feeds were initiated, in contrast to the previous study [23], to maintain adequate nutritional and fluid intake. This result suggests that a longer period of TPN prior to initiation of enteral feeds is protective against NEC by allowing time for maturation of the preterm intestine. However, this finding is in contrast to a review of seven randomized controlled trials that showed no evidence that delaying the introduction of enteral feeding reduces the risk of developing NEC in very preterm or VLBW infants [16].

Our second finding showed that gradual advancement of enteral feeding while providing PN did not prevent the development of NEC based on the overall histological and clinical NEC scores. However, there was a lower colonic histological NEC severity score in the EG vs. EA group. This combined with a significantly lower expression of several proinflammatory genes in EG vs. EA pigs also suggest that NEC was less severe with gradual feeding advancement even though the incidence was not statistically significant. This approach is designed to allow more time for gut maturation prior to introducing a potential trigger for gut injury (high-volume enteral feeds). This finding is in accordance with a review of five randomized controlled trials suggesting that slow advancement of enteral feeds does not reduce the risk of developing NEC in very preterm or VLBW infants compared to faster advancement [17]. Thus, our results do not show strong or conclusive evidence (based on NEC incidence and severity) that gradual feeding advancement is beneficial, but perhaps the differences between EG vs. EA are subtle and require larger animal numbers to establish statistical significance. Another clinically important comparison between these treatment groups would be to compare the LA vs. EG groups. The measures of clinical NEC incidence were similar between LA vs. EG. However, the clinical and histological NEC severity scores tended to be lower in the LA vs. EG groups. This suggests that delaying the introduction of enteral feeding is protective regardless of whether you begin feeds early and advance rapidly or slowly.

Despite the increasing knowledge of the pathophysiology of NEC, the exact etiology of NEC development in preterm infants remains unclear and is still under investigation but the major risk factors include GIT immaturity, enteral feeding, and bacterial colonization [3], [24]. The interaction between an immature intestinal immune function and bacterial colonization seems to be a key trigger in NEC pathogenesis. Some key inflammatory mediators activated by bacterial colonization include tumor necrosis factor-alpha (TNF-α), platelet activating factor (PAF), Toll-like receptor 4 (TLR4) and the interleukins IL-1β, IL-6 and IL-8 [7]. TLR4 specifically recognizes the bacterial cell wall component lipopolysaccharide (LPS) on gram-negative bacteria [25] and studies in infants and rodents have linked increased TLR4 expression with NEC pathogenesis [26], [27]. Thus, during the onset of NEC, the activation of TLR4-MyD88 signaling and the transcription factor nuclear factor kappa B leads to increased expression of proinflammatory genes such as IL-1β, IL-6 and TNF-α [28]. In the current study, we found no difference in ileal TNF-α expression between EA vs. LA (Experiment 1) and EA vs. EG (Experiment 2). In contrast, we found that TLR4 expression was lower in the EG group vs. EA group (Experiment 2), but not different between the EA and LA groups (Experiment 1). However, ileal IL-1β and IL-6 expression were lower in the LA group (Experiment 1) and the EG group (Experiment 2) compared to the EA group. Taken together, these findings suggest that the protective effects of delayed introduction or gradual advancement of enteral feeding resulted from lower intestinal inflammation.

Two members of the S100 protein family, S100A8 and S100A9, have been identified as important damage-associated molecular pattern (DAMP) ligands released by activated phagocytes and recognized by TLR4 on monocytes [29]. S100A8 (also termed MRP8) and S100A9 (MRP14) exist mainly as a S100A8/S100A9 heterodimer termed calprotectin [30]. Fecal calprotectin concentration has been reported to be increased in preterm infants with NEC and may be an accurate marker for early diagnosis of NEC [31], [32] Consistent with these reports, we found that ileal S100A9 expression was lower in both the LA and EG groups vs. EA group (Experiments 1 and 2). In addition, our results showed that bloody stool was the most specific predictive clinical sign for NEC, whereas the presence of abdominal distention was the most sensitive predictive clinical signs for NEC [3], [33].

Another important finding in this study was that pigs fed hydrolyzed vs. intact protein formula had improved gastric transit evident by decreased stomach content weights at the time of death, without an increased risk of NEC. It is important to note that the hydrolyzed protein formula contained intact whey protein, but the casein was hydrolyzed to limit the formation of clotting in the stomach. The slower gastric emptying rate in the pigs fed intact protein formula was also evident in increased abdominal distension and respiratory distress without any evidence of NEC; this adverse effect of intact protein on stomach clotting and respiratory distress is why we used only hydrolyzed protein in Experiment 2. We suspect this finding is due to increased gastric clotting and slowed digestion of casein proteins present in intact protein formula as previously described in human adults [34], neonatal pigs [35], and preterm infants [36]. Studies have shown that partially hydrolyzed protein formula was associated with improved feeding intolerance (i.e. more rapid gastric emptying, shorter gastrointestinal times, and shorter time to establish full enteral feeds) compared to intact protein formula in preterm infants [36], [37]. Thus, the current results support the concept of feeding formula with hydrolyzed casein protein as a means to improve gastric emptying and feeding tolerance, without adversely increasing the risk of NEC.

In conclusion, our results in the preterm piglet provides evidence for the prevention and reduction in severity of NEC via delayed introduction of enteral feeding while providing adequate nutrition and fluid support through PN. The results from the current study do not support the approach of gradual vs. abrupt introduction of large volume enteral feeding as a means to prevent NEC. Despite our results in preterm piglets and meta-analysis of clinical studies showing no benefit of gradual feeding in prevention of NEC, this practice continues to be widespread clinically due to the limitations associated with feeding intolerance per se. In addition, this study supports the finding in human studies that hydrolyzed vs. intact protein formula improves gastric transit without increasing the risk of NEC and thus may be useful for minimizing feeding intolerance in preterm infants. The fact that our current results in preterm piglets are consistent with two consensus findings from clinical studies further highlights the relevance of the preterm pig model for investigating how improved nutrition and management practices can be translated into clinical care of human preterm infants.

## Supporting Information

Figure S1 **NEC clinical symptoms occurrence in Experiment 1.** (A) Diarrhea, (B–C) abdominal distension, and (D–E) respiratory distress occurrence (A, B, and D) in pigs in the EA vs. LA group and (C, E) in pigs from the EA group fed either an intact (Intact protein) or hydrolyzed protein (Hydrolyzed protein) formula. EA, early abrupt; LA, late abrupt, TRT, treatment; Nutr, nutrition.
(PDF)

Figure S2 **Intestinal cross sections from Experiment 1.** Histological cross sections stained with H&E of the (A) jejunum and (B) colon from pigs fed either an intact or hydrolyzed protein formula without NEC (No NEC) or that had developed NEC (NEC). All images are presented at 10X magnification. EA, early abrupt; LA, late abrupt.
(PDF)

Figure S3 **NEC clinical symptoms occurrence in Experiment 2.** (A) Gastric residuals, (B) diarrhea, and (C) abdominal distension occurrence in pigs. EA, early abrupt; LA, late abrupt, TRT, treatment.
(PDF)

Figure S4 **Intestinal cross sections from Experiment 2.** Histological cross sections stained with H&E of the (A) jejunum and (B) colon in pigs without NEC (No NEC) or that had developed NEC (NEC). All Images are presented at 10X magnification. EA, early abrupt; EG, early gradual.
(PDF)

Table S1 Enteral Formula Composition.
(DOCX)

Table S2 Forward and reverse primer sequences for porcine gene quantification by RT-qPCR.
(DOCX)
