# Supplementary figures and images for: Delayed Initiation but Not Gradual Advancement of Enteral Formula Feeding Reduces the Incidence of Necrotizing Enterocolitis (NEC) in Preterm Pigs
Source: PLoS One. 2014 Sep 19;9(9):e106888. doi: 10.1371/journal.pone.0106888 (PMC4169518; doi:10.1371/journal.pone.0106888)

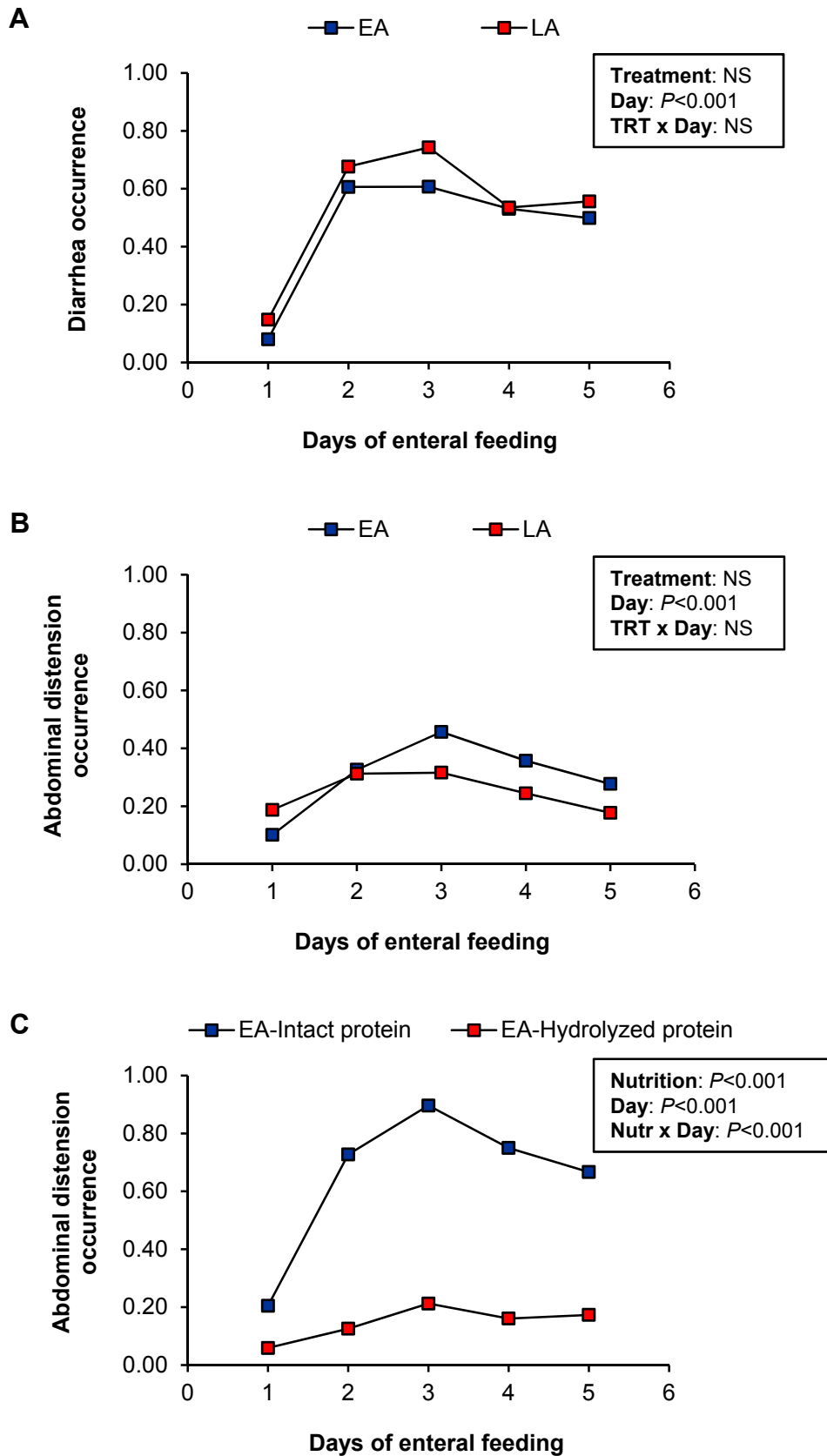

Figure S1

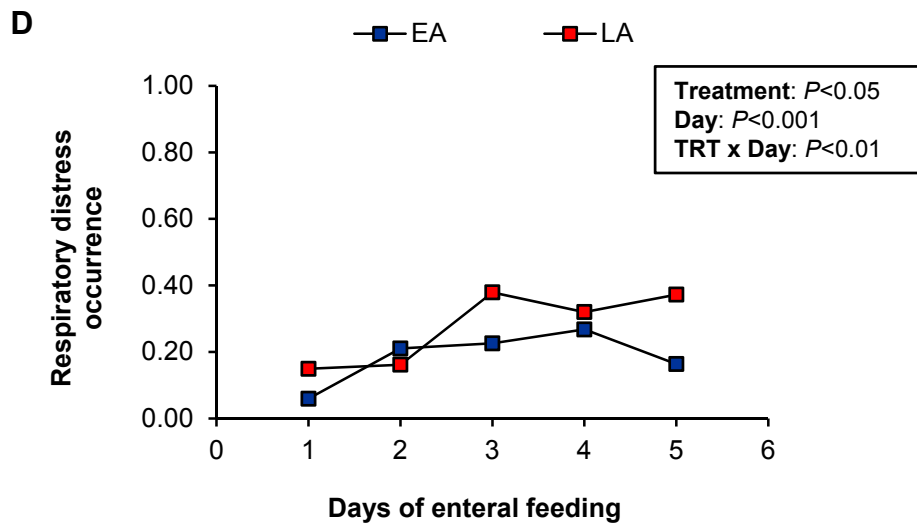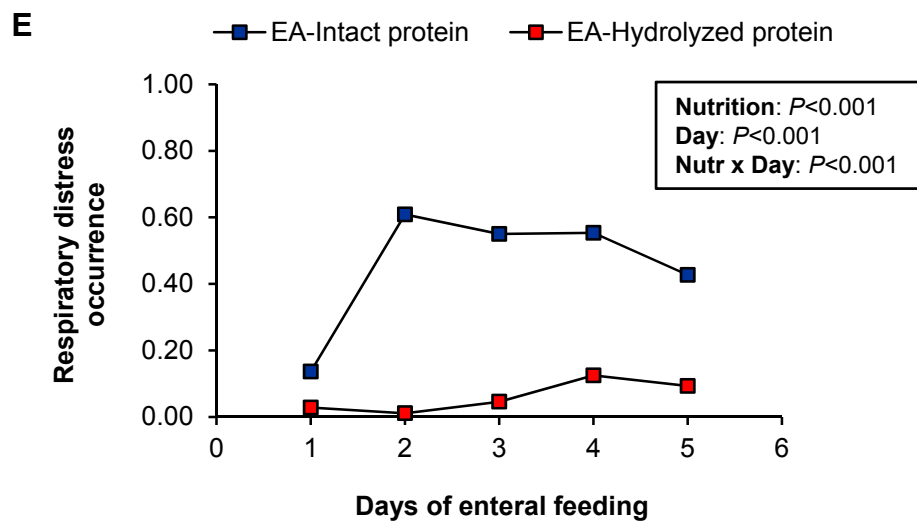

Figure S1

Supplement: Figure S1 — NEC clinical symptoms occurrence in Experiment 1. (A) Diarrhea, (B–C) abdominal distension, and (D–E) respiratory distress occurrence (A, B, and D) in pigs in the EA vs. LA group and (C, E) in pigs from the EA group fed either an intact (Intact protein) or hydrolyzed protein (Hydrolyzed protein) formula. EA, early abrupt; LA, late abrupt, TRT, treatment; Nutr, nutrition. (PDF) [file pone.0106888.s001.pdf]

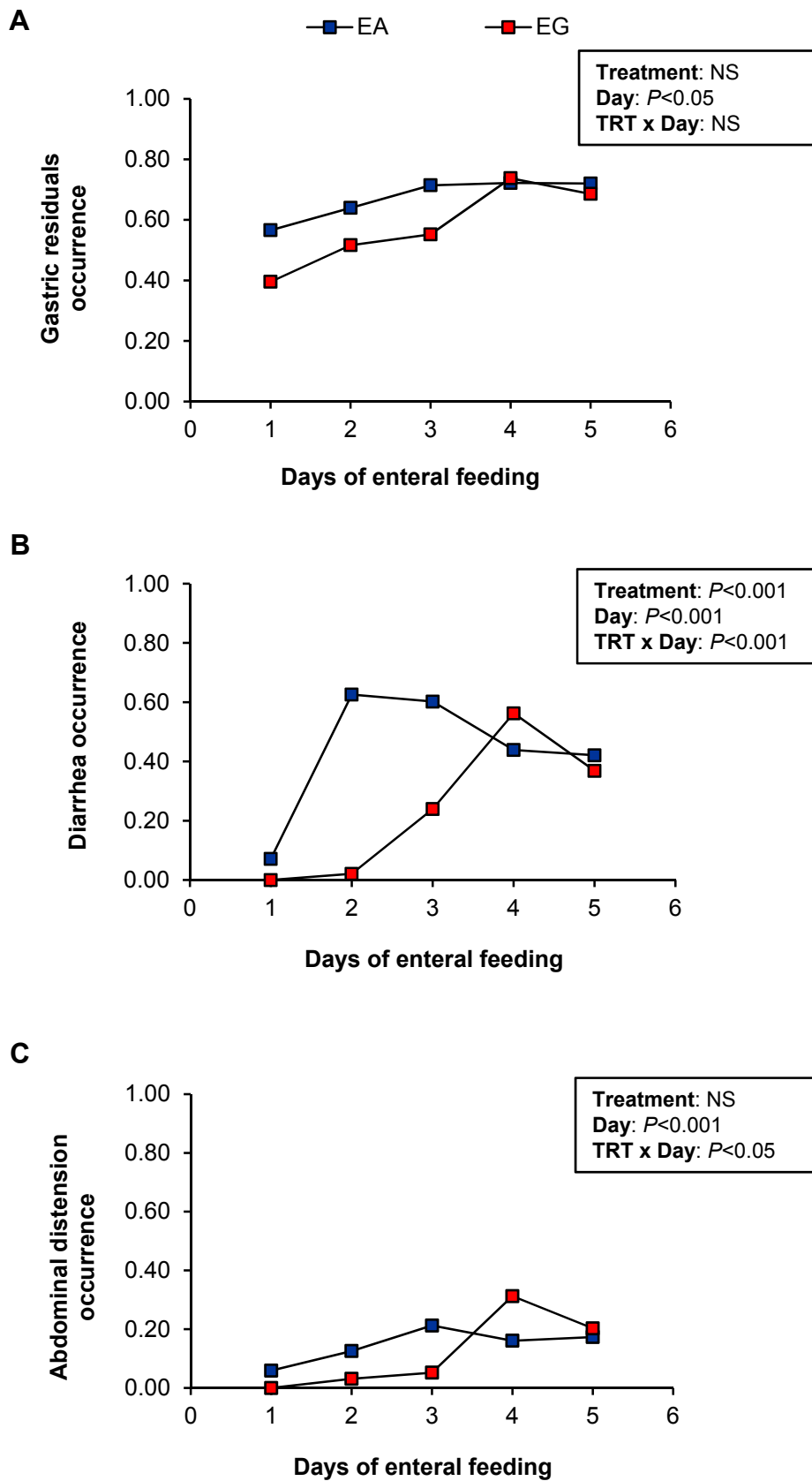

Figure S3

Supplement: Figure S3 — NEC clinical symptoms occurrence in Experiment 2. (A) Gastric residuals, (B) diarrhea, and (C) abdominal distension occurrence in pigs. EA, early abrupt; LA, late abrupt, TRT, treatment. (PDF) [file pone.0106888.s003.pdf]

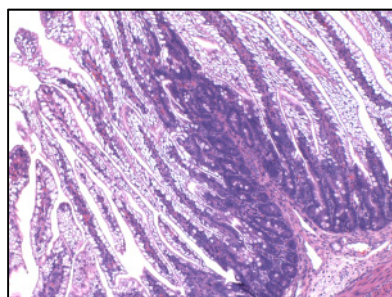

**EA-No NEC**

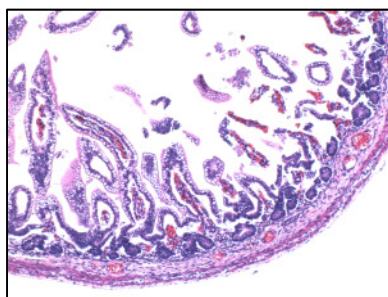

**EA-NEC**

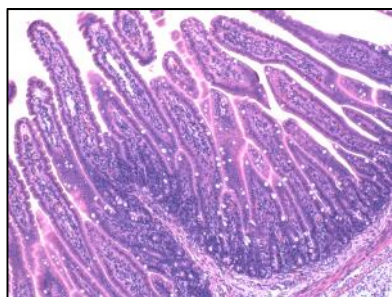

**EG-No NEC**

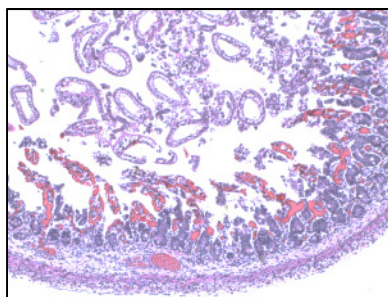

**EG-NEC**

**Panel A**

**Figure S4**

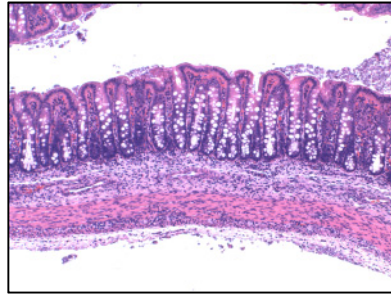

**EA-No NEC**

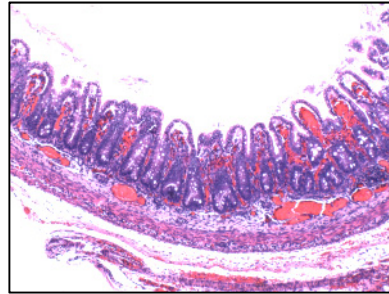

**EA-NEC**

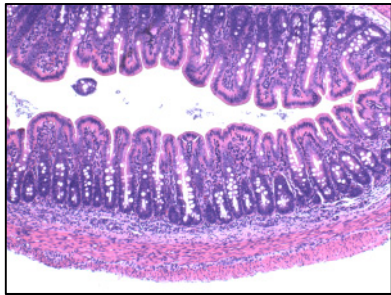

**EG-No NEC**

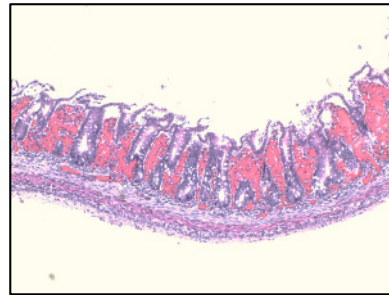

**EG-NEC**

**Panel B**

**Figure S4B**

Supplement: Figure S4 — Intestinal cross sections from Experiment 2. Histological cross sections stained with H&E of the (A) jejunum and (B) colon in pigs without NEC (No NEC) or that had developed NEC (NEC). All Images are presented at 10X magnification. EA, early abrupt; EG, early gradual. (PDF) [file pone.0106888.s004.pdf]
