# Supplementary material for: Delayed Initiation but Not Gradual Advancement of Enteral Formula Feeding Reduces the Incidence of Necrotizing Enterocolitis (NEC) in Preterm Pigs
Source: PLoS One. 2014 Sep 19;9(9):e106888. doi: 10.1371/journal.pone.0106888 (PMC4169518; doi:10.1371/journal.pone.0106888)
Supplement: Figure S2 — Intestinal cross sections from Experiment 1. Histological cross sections stained with H&E of the (A) jejunum and (B) colon from pigs fed either an intact or hydrolyzed protein formula without NEC (No NEC) or that had developed NEC (NEC). All images are presented at 10X magnification. EA, early abrupt; LA, late abrupt. (PDF) [file pone.0106888.s002.pdf]

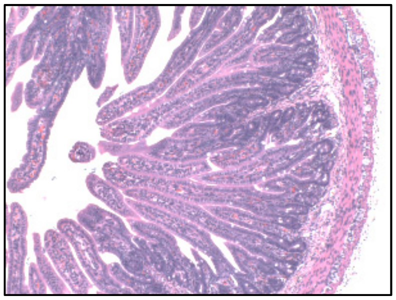

EA-Intact protein-No NEC

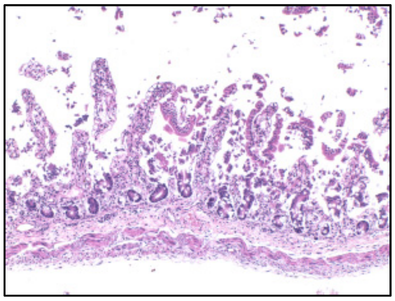

EA-Intact protein-NEC

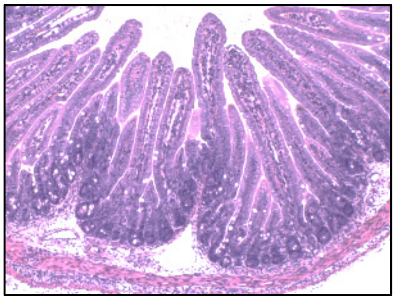

EA-Hydrolyzed protein-No NEC

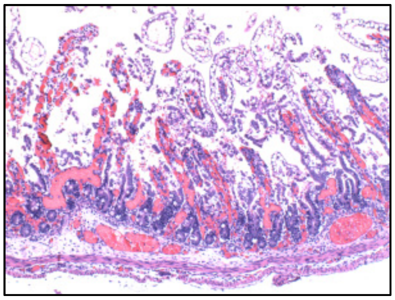

EA-Hydrolyzed protein-NEC

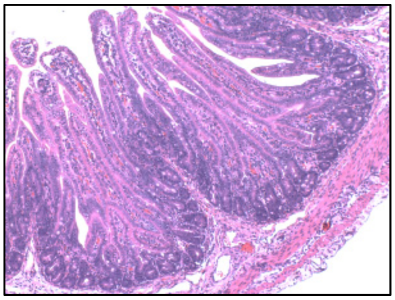

LA-Intact protein-No NEC

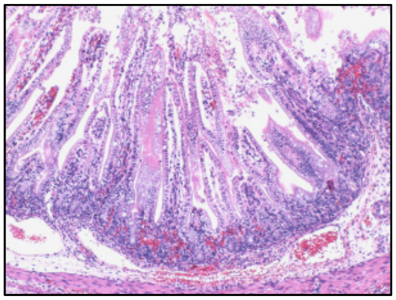

LA-Intact protein-NEC

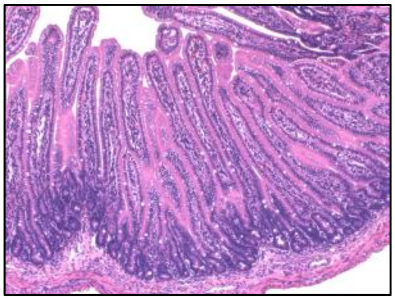

LA-Hydrolyzed protein-No NEC

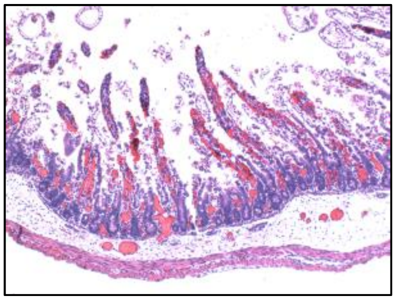

LA-Hydrolyzed protein-NEC

Panel A

Figure S2

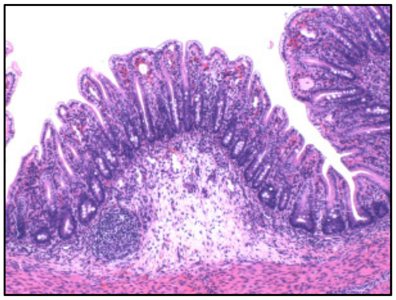

EA-Intact protein-No NEC

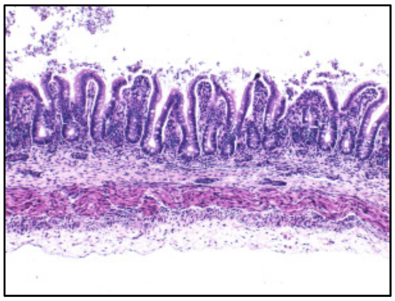

EA-Intact protein-NEC

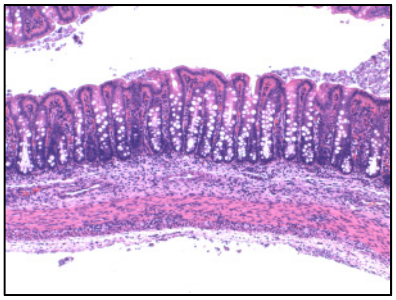

EA-Hydrolyzed protein-No NEC

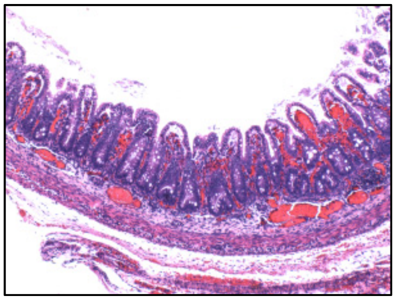

EA-Hydrolyzed protein-NEC

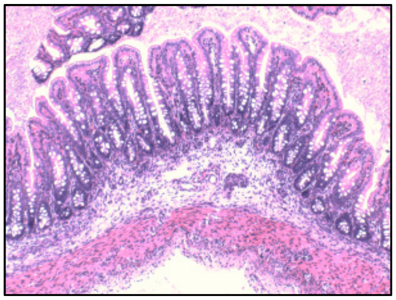

LA-Intact protein-No NEC

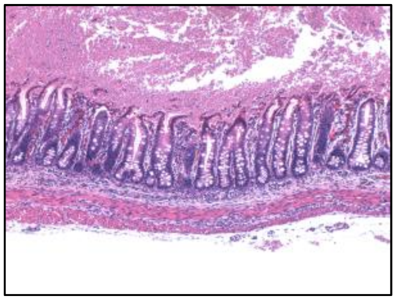

LA-Intact protein-NEC

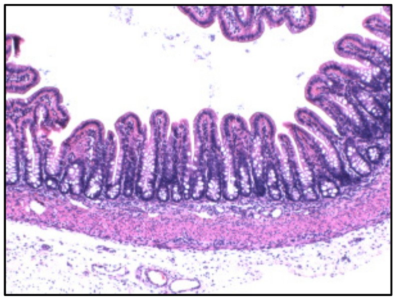

LA-Hydrolyzed protein-No NEC

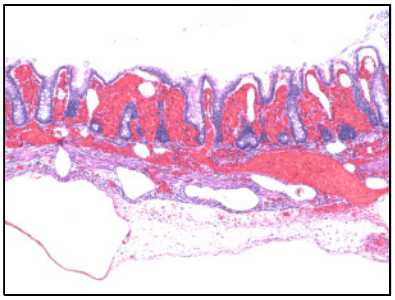

LA-Hydrolyzed protein-NEC

Panel B

Figure S2
