# Supplementary material for: Delayed Initiation but Not Gradual Advancement of Enteral Formula Feeding Reduces the Incidence of Necrotizing Enterocolitis (NEC) in Preterm Pigs
Source: PLoS One. 2014 Sep 19;9(9):e106888. doi: 10.1371/journal.pone.0106888 (PMC4169518; doi:10.1371/journal.pone.0106888)
Supplement: Table S1 — Enteral Formula Composition. (DOCX) [file pone.0106888.s005.docx]

**Supporting Information**

Table S1. Enteral Formula Composition.

|  | **Intact protein** | **Hydrolyzed protein** |
| --- | --- | --- |
|  | *g/L* | |
| **Protein** | **65.5** | **66.4** |
| Milk protein isolate | 34.1 |  |
| Casein hydrolysate |  | 28.7 |
| Whey protein isolate | 31.4 | 37.7 |
| **Fat** | **59.0** | **59.0** |
| Medium-chain triglyceride oil | 24.7 | 24.7 |
| Soybean oil | 18.2 | 18.2 |
| High oleic sunflower oil | 16.1 | 16.1 |
| **Carbohydrate** | **59.2** | **60.1** |
| Corn syrup solids | 59.2 | 60.1 |
