# Supplementary material for: Delayed Initiation but Not Gradual Advancement of Enteral Formula Feeding Reduces the Incidence of Necrotizing Enterocolitis (NEC) in Preterm Pigs
Source: PLoS One. 2014 Sep 19;9(9):e106888. doi: 10.1371/journal.pone.0106888 (PMC4169518; doi:10.1371/journal.pone.0106888)
Supplement: Table S2 — Forward and reverse primer sequences for porcine gene quantification by RT-qPCR. (DOCX) [file pone.0106888.s006.docx]

Supporting Information

Table S2. Forward and reverse primer sequences for porcine gene quantification by RT-qPCR.

| **Genes** | **Forward and reverse primer sequences** | **Accession number** |
| --- | --- | --- |
| **β-Actin** | 5’-GGACCTGACCGACTACCTCA-3’ | DQ452569 |
|  | 5’-GCGACGTAGCAGAGCTTCTC-3’ |  |
| **IL-1 β** | 5’-AGGCAGATGGTGTCTGTCATC-3’ | NM_214055 |
|  | 5’-AGGATGATGGGCTCTTCTTCAAA-3’ |  |
| **IL-6** | 5’-TCTGGGTTCAATCAGGAGACC-3’ | NM_214399 |
|  | 5’-CTAATCTGCACAGCCTCGAC-3’ |  |
| **S-100A9** | 5’-ATGGAATGCAGCATAGAAAC-3’ | NM_001177906.1 |
|  | 5’-TCTTCTGCTTCTTGAGAAAGT-3’ |  |
| **TLR4** | 5’-TGGATTTATCCAGATGTGAAA-3’ | NM_001113039.1 |
|  | 5’-CTGTAAACTTGGTAGTCCAG-3’ |  |
| **TNF-α** | 5’-GGCCCAAGGACTCAGATCAT-3’ | JF831365 |
|  | 5’-TGAGGTACAGCCCATCTGTC-3’ |  |
